# Effect of a 1-week, eucaloric, moderately high-fat diet on peripheral insulin sensitivity in healthy premenopausal women

**DOI:** 10.1136/bmjdrc-2015-000100

**Published:** 2015-07-16

**Authors:** Natalia M Branis, Marjan Etesami, Ryan W Walker, Evan S Berk, Jeanine B Albu

**Affiliations:** 1 New York Obesity Research Center, St. Luke's Roosevelt Hospital Center, New York, New York, USA; 2 Division of Endocrinology, Diabetes and Metabolism, University of Rochester School of Medicine and Dentistry, Rochester, New York, USA; 3 Department of Medicine, Palomar Medical Center, Escondido, California, USA; 4 The Charles Bronfman Institute for Personalized Medicine, Icahn School of Medicine at Mount Sinai, New York, New York, USA; 5 Nutrition Performance Unit, Glaxo Smith Kline, Parsippany, New Jersey, USA; 6 Division of Endocrinology, Diabetes and Nutrition, Mount Sinai St Luke's and Mount Sinai Roosevelt Hospitals, Icahn School of Medicine at Mount Sinai, New York, New York, USA

**Keywords:** Insulin Resistance, Dietary Fat, Euglycemic Clamp, African American(s)

## Abstract

**Objectives:**

To determine whether a weight-maintaining, moderate (50%) high-fat diet is deleterious to insulin sensitivity in healthy premenopausal women.

**Design/setting/participants:**

23 African-American and non-Hispanic white, healthy, overweight, and obese premenopausal women recruited in New York City, USA, fed either a eucaloric, 1-week long high-fat (50% of total Kcal from fat) diet or a eucaloric, 1-week long low-fat (30% of total Kcal from fat) diet, assigned in a randomized crossover design.

**Main outcome measures:**

Peripheral insulin sensitivity and metabolic flexibility during a euglycemic hyperinsulinemic (80 mU/m^2^/min) clamp measured during the follicular phase of the menstrual cycle, at the end of each diet period.

**Results:**

Peripheral insulin sensitivity (mg kg/fat-free mass/min (µU/mL)×10^−1^) was not decreased after the high-fat diet vs the low-fat diet (0.09±0.01 vs 0.08±0.01, p=0.09, respectively) in the combined group of African-American and white women, with no significant diet by race interaction (p=0.6). Metabolic flexibility (change in substrate utilization, ΔNPRQ, in response to insulin during the clamp) was similarly unaltered by the diet (0.12±0.01 vs 0.11, p=0.48, for the high-fat diet vs the low-fat diet, respectively) in the combined group of women, with no significant diet by race interaction (p=0.9). African–American women had a lower insulin clearance compared with the white women, regardless of the diet (p<0.05).

**Conclusions:**

We conclude that a short term (1 week), moderate (50%), eucaloric high-fat diet does not lower peripheral insulin sensitivity in healthy, overweight and obese premenopausal women.

**Key messages**

- There is controversy over whether a eucaloric, moderately high-fat (50%) diet vs a lower fat (30%) diet induces insulin resistance in overweight and obese women; substituting fat for carbohydrates to a moderate degree (50% vs 30%) in a weight-maintaining diet is not deleterious for peripheral insulin action in healthy overweight and obese women, at least in the short term (1 week).
- Similarly, metabolic flexibility (the ability to suppress fat oxidation by insulin during a hyperinsulinemic clamp) is not affected by a higher (50%) vs a lower fat (30%) eucaloric diet in healthy overweight and obese women, at least in the short term (1 week).
- African–American women are more insulin resistant and have lower rates of postabsorptive fat oxidation than similar white women, as we have previously reported, but we did not find that a moderately higher fat diet (50%) compared to a lower fat diet (30%) adversely affects their peripheral insulin action or ability to suppress fat oxidation during a high-dose insulin clamp.

## Introduction

The role of the macronutrient composition of the diet with regard to the carbohydrate-to-fat ratio in the treatment of obesity and diabetes prevention has been only partially elucidated. While a low-fat (LF) diet was the mainstay for the diabetes prevention program^1^ and is the basis for the 2010 Dietary Guidelines for Americans,^2^ hypocaloric diets of both high-fat (HF) and LF compositions have been effective for weight loss.^3^ Epidemiologically, higher total fat intake was associated with higher rates of progression to type 2 diabetes in the San Luis Valley Diabetes study^4^; however, two other large population-based studies in women (Iowa Women's and Nurses’ Health studies) did not replicate these findings.^5^ ^6^

Whether increasing the fat-to-carbohydrate ratio of a eucaloric, weight-maintaining diet decreases insulin sensitivity is controversial, particularly in women.^7–12^ One study in women has shown a decrease in insulin sensitivity, measured by a frequently sampled intravenous glucose tolerance test (FSIVGTT), after 3 weeks of an HF diet compared to a LF diet in healthy premenopausal African–American and non-Hispanic (NH) white participants.^13^ However, other work has demonstrated that peripheral insulin sensitivity, measured by the euglycemic hyperinsulinemic clamp, does not decrease after eucaloric HF diets of various durations (6 days and up to 3 weeks) in lean or obese men^7–10^ or combined groups of lean men and women.^11^ ^12^ Metabolic flexibility (the ability to suppress fat oxidation during the euglycemic hyperinsulinemic clamp) has been closely associated with insulin sensitivity^14^ ^15^ and decreased in response to a HF diet in men,^8^ ^9^ yet this has never been studied in women.

Therefore, we aimed to determine whether insulin sensitivity measured during a euglycemic hyperinsulinemic clamp will be deleteriously affected by a 1 week, eucaloric HF (50% total Kcal from fat) diet in African–American and non-Hispanic white, healthy, premenopausal, overweight and obese women. In addition, we determined the effect of the diets on metabolic flexibility during the clamps. We and others have previously reported lower peripheral insulin sensitivity^16–20^ differences in muscle adipose tissue distribution^19^ and lower systemic rates of fat oxidation in African–American vs non-Hispanic white women.^15^ ^21^ ^22^ Therefore, we also examined any race differences in substrate utilization during the clamps.

## Research design and methods

### Subjects

Twenty-three healthy premenopausal (25–45 years) overweight and obese (body mass index, BMI 25–40 kg/m^2^) women (11 African–American and 12 non-Hispanic white) participated in the study. Participants were included if they reported all four grandparents to be of African or Caucasian descent, had regular menstrual cycles, and were without diabetes according to an oral glucose tolerance test (75 g glucose load). Self-reported use of any medications (including contraceptive pills), smoking within the past 6 months, and consumption of >2 oz. ethanol/day were exclusionary. All participants signed consent forms approved by the St. Luke's-Roosevelt Institute for Health Sciences Institutional Review Board.

### Study design

In a randomized crossover design, participants consumed a LF (30% fat, 50% carbohydrate and 20% protein) or a HF (50% fat, 30% carbohydrate and 20% protein) weight-maintaining diet for seven consecutive days as per the protocol we had previously published.^15^ On the morning of day 8 after an overnight admission to the Clinical Research Center at St. Luke's-Roosevelt Hospital Center, insulin sensitivity and substrate utilization were measured before and during a euglycemic hyperinsulinemic clamp. There was a minimum 2-week washout period between diets. All measurements were conducted during the follicular phase of the menstrual cycle.

### Dietary protocol

All study participants completed dietary surveys indicating foods they liked and disliked. Eucaloric, weight-maintaining diets were constructed from food items available commercially with known macronutrient and caloric composition. Food item caloric content and macronutrient composition were verified using Nutritionist IV (V.2.0, Nsquared Commuting Co, Salem,Oregon, USA). Total daily calories for weight maintenance were calculated based on resting metabolic rate measured by indirect calorimetry in a fasting state (Horizon metabolic Cart or V-Max29; Sensor Medics, Yorba Linda, California, USA) and multiplied by an activity factor (1.5). Diets were matched in distribution of fat calories with equal parts of saturated fat, monounsaturated fat and polyunsaturated fat. Participants were provided with a 7-day food supply to consume at home. Dietary compliance was assured through weight stability measurements and adjustments were planned for a weight change of more than 1 kg.

### Insulin sensitivity

Following an overnight fast, a three-hour euglycemic hyperinsulinemic clamp (80 mU/m^2^/min) was performed. We used a high-dose insulin clamp to measure the effect of the diet on peripheral insulin sensitivity in African–American vs non-Hispanic white women as we sought differences between races as well. We, as others, have previously reported lower peripheral insulin sensitivity^16–20^ in African–Americans vs non-Hispanic whites. Blood samples were collected at 10 min intervals during the postabsorptive and steady state of hyperinsulinemic euglycemic clamp, immediately centrifuged, aliquoted and frozen at −70°C. Insulin was measured by RIA (Linco Research, St. Charles, Missouri, USA), glucose was measured by the Beckman glucose analyzer (Beckman, Fullerton, California, USA) and non-esterified fatty acids (NEFA) were measured by the enzymatic colorimetric method (Wako Chemicals USA, Richmond, Virginia, USA). NEFA suppression was calculated as the difference between the NEFA levels at steady state and the postabsorptive NEFA levels divided by the postabsorptive NEFA levels times 100 (percentage). Insulin clearance was calculated according to DeFronzo^23^ as the ratio of the difference in insulin concentration between the post-absorptive and steady states and the rate of insulin infusion during the clamp study, which was assumed to be 80 mU/m^2^/min for all participants. Insulin sensitivity was calculated as M/I using the glucose disposal rate M (mg kg/fat-free mass (FFM)/min) and insulin concentration in the hyperinsulinemic steady state I (µU/mL).

### Indirect calorimetry

Oxygen consumption (VO_2_) and carbon dioxide production (VCO_2_) were measured using a ventilated hood in the postabsorptive and hyperinsulinemic steady states of the euglycemic clamp. In both states, the participants were supine and awake. Substrate oxidation rates were calculated using Frayn's equations,^24^ and non-protein respiratory quotient (NPRQ) was calculated as a ratio of VCO_2_ to VO_2_. Metabolic flexibility was estimated as a change in NPRQ (ΔNPRQ) between the postabsorptive and hyperinsulinemic steady states.

### Statistics

All data are reported as mean±SEM as noted. All variables were checked for normality of distribution; only fasting triglycerides were log transformed for analyses (log10). Statistical comparison of participant characteristics by race was performed using the independent t test (table 1). Selection and confounding biases were controlled for by symmetrical case-crossover methodology with identical length of exposure to the LF and HF diets. While the participants were unaware of the diet composition, there was no allocation concealment from the investigators.

**Table 1:** Participants’ characteristics

Characteristics	All participants (23)	African–American (11)	Non-Hispanic white (12)
Age (years)	33.61±1.18	32.27±1.80	34.83±1.54
BMI (kg/m^2^)	29.65±0.90	28.80±1.12	30.42±1.39
Percentage of fat*	42.21±1.72	40.03±2.57	44.22±2.24
FM (kg)*	33.83±2.14	31.32±2.98	36.13±3.02
FFM (kg)*	44.85±0.91	45.39±1.02	44.35±1.51
Fasting triglycerides (mmol/L)	0.85±0.11	0.82±0.16	0.88±0.16
Fasting HDL (mmol/L)	0.90±0.04	0.90±0.07	0.91±0.07

Data are mean±SEM.

There were no significant differences in subject characteristics between African–American and non-Hispanic white women (p range 0.23–0.91).

*Determined by dual X-ray absorptiometry (DXA).

BMI, body mass index; HDL, high-density lipoprotein; FM, fat mass; FFM, fat-free mass.

**Figure 1:**
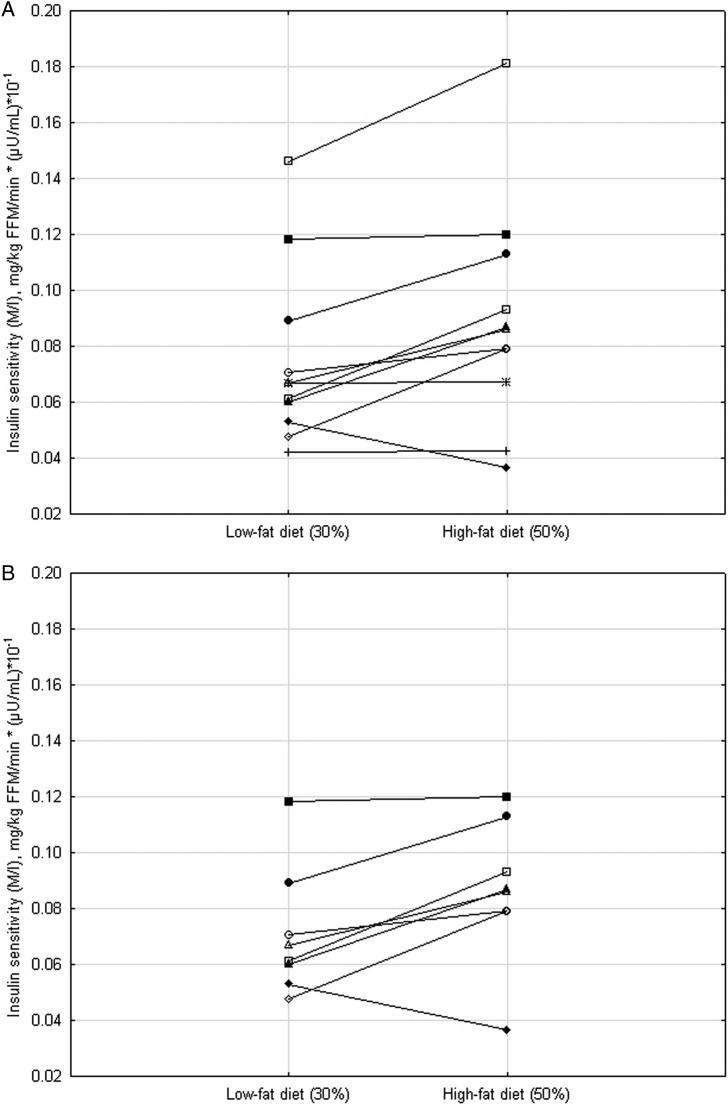
(A–B) Insulin sensitivity after 7 days of a eucaloric low-fat diet (30%) or high-fat (50%) diet in non-Hispanic white (panel a) and African–American (panel b) women.

**Figure 2:**
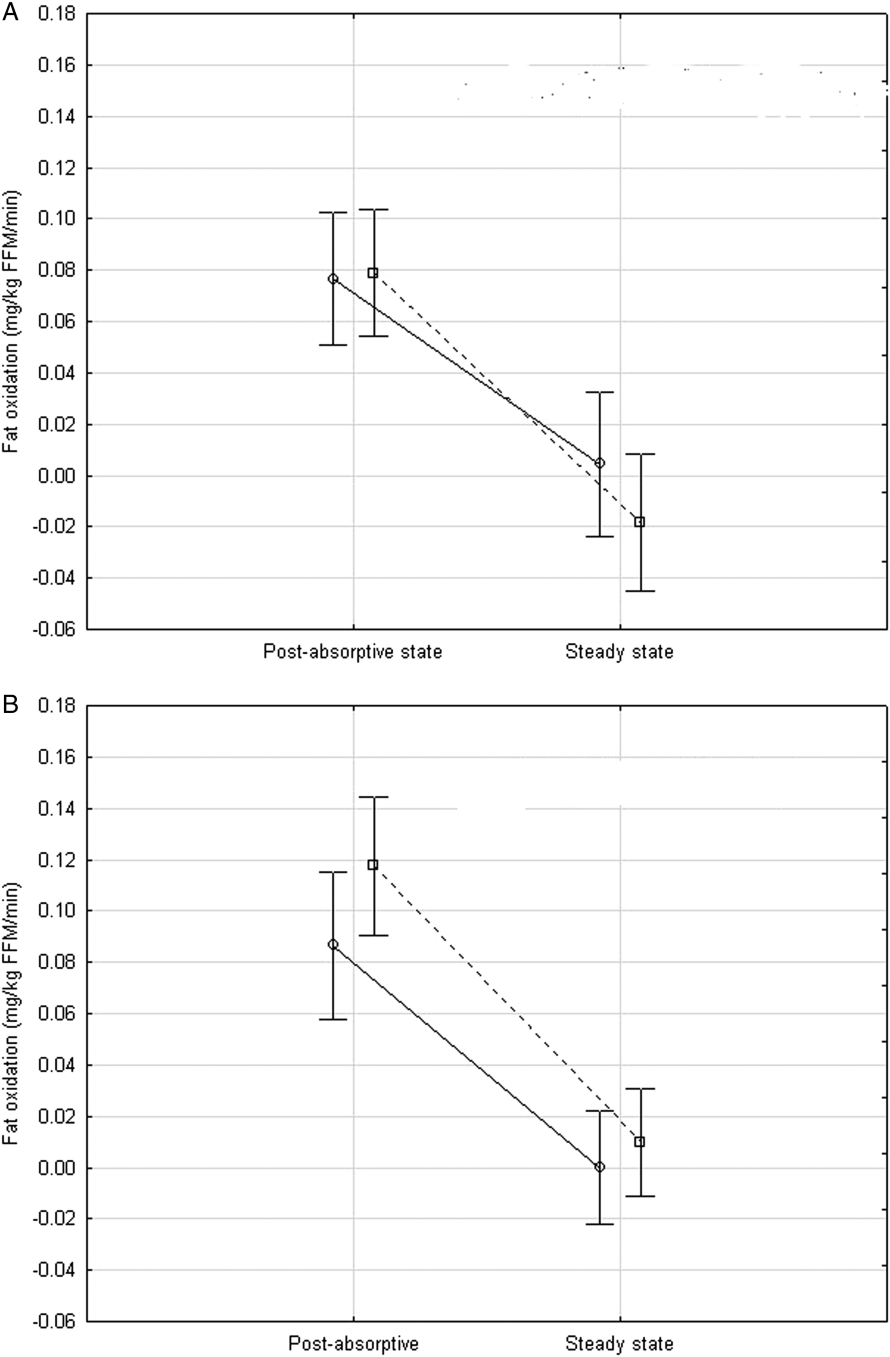
(A–B) Differences in fat oxidation during the hyperinsulinemic euglycemic clamp between African–American and non-Hispanic white women after 7 days of a eucaloric low-fat diet (30%) (A) OR after 7 days of a eucaloric high-fat diet (50%) (B). Open circles, solid lines=African–American women. Open squares, dotted lines=non-Hispanic white women.

Analysis of variance and multivariate analysis of variance (ANOVA/MANOVA) were used to determine the effects of diet (LF vs HF, repeated measures, within effect) and to compute diet by race interactions (African–Americans vs non-Hispanic whites, between effect) from measures in the postabsorptive state and during the steady state of the clamp (glucose, NEFA and insulin levels, substrate utilization, NEFA suppression, insulin clearance, insulin sensitivity and metabolic flexibility), as shown in table 2 and figures 1A, B and 2A, B. Only data from women who completed either one of the dietary interventions (LF or HF diet) were used for analysis. A power analysis was performed for the effect of diet on peripheral insulin sensitivity, using initial pilot data (first seven participants of the study), which yielded a large effect size, Cohen's d=0.81 (M/I change between diets mean±SD, 0.014262±0.017618) from which the required sample size for 2 tailed α=0.05, power=0.80 was calculated to be n=15.

**Table 2:** Effect of diet on insulin sensitivity and substrate utilization (repeated measures)

	All subjects (19)		p Values
	Low-fat diet	High-fat diet	
Postabsorptive			
Glucose	5.23±0.07	5.18±0.08	0.49
Insulin	71.78±4.90	71.22±5.53	0.77
NEFA	0.55±0.03	0.53±0.03	0.47
NPRQ*	0.90±0.01	0.87±0.01	0.12
Steady state			
Glucose	5.73±0.08	5.67±0.08	0.52
Insulin	1331.78±54.58	1362.86±73.12	0.41
NEFA	0.04±0.00	0.04±0.00	0.79
NPRQ*	1.01±0.01	0.99±0.01	0.15
Ins. clearance	455.73±20.29	453.15±24.69	0.67
GDR†	14.09±0.78	15.51±0.84	0.02
M/I	0.08±0.01	0.09±0.01	0.09
NEFA % supp.	92.90±0.67	92.66±0.73	0.72
ΔNPRQ*	0.11±0.01	0.12±0.01	0.48

Data are mean±SEM. Glucose in mmol/L; insulin in pmol/L; non-esterified fatty acids (NEFA) in mmol/L; non-protein respiratory quotient (NPRQ); insulin clearance (Ins. Clear. in mL m^2^/BSA/min; glucose disposal rate (GDR) in mg kg/FFM/min; insulin sensitivity (M/I)=GDR/steady-state insulin; % of non-esterified fatty acids suppression (NEFA %); metabolic flexibility (ΔNPRQ).

*Data shown for 15 participants (7 African–American and 8 non-Hispanic white women) who completed substrate utilization measures for the LF and HF diets (repeated measures).

†p=0.02 for the effect of diet on GDR (higher after the HF diet); other effects of diet were not significant (p range 0.09–0.79).

BSA, body surface area; GDR, glucose disposal rate; HF, high-fat diet; LF, low-fat diet; NPRQ, non-protein respiratory quotient; NEFA, non-esterified fatty acids.

A general linear model was used to determine whether there were any race differences in response to insulin during the euglycemic hyperinsulinemic clamps, for the LF and HF diets separately (figure 2A and B). Both differences by race and any interactions by race in the effect of insulin during the clamps were computed for the LF and HF diets separately. The difference by race in steady-state insulin levels was also determined after adjusting for the postabsorptive insulin level (as a covariate). No other covariates were included in the analyses.

A p value less than 0.05 was considered statistically significant. Statistical analysis was performed using Statistica (V.10.0, Tulsa, Oklahoma, USA).

## Results

Participant characteristics are shown in table 1. Twenty-three premenopausal (age 33.61±1.18 years) overweight (BMI 29.65±0.90 kg/m^2^) women participated in the study. Eight of 11 African–American and 11 out of 12 white women completed insulin sensitivity measurements after both the LF and HF diet periods (repeated measures). Additionally, one African–American woman completed the studies only after the LF diet condition and three women (2 African–Americans and 1 white) completed the studies only after the HF diet condition. For personal reasons, they did not participate in the second dietary period. There were no statistically significant differences in age, BMI, body composition measurements, fasting triglycerides and high-density lipoprotein (HDL)-cholesterol levels between the two races (table 1) or in the subgroups which had repeated measures (not shown).

For the 19 participants who had repeated measures, the effect of diet on insulin sensitivity and metabolic flexibility (ΔNPRQ during the clamp) are shown in table 2. There were no significant diet by race interactions on any of the variables (p range 0.31 to 1.0); thus, the main effects of diet are presented here. Insulin sensitivity computed as the glucose disposal rate per kg of FFM and divided by the steady-state insulin level (M/I) was not significantly decreased by the diet in the African–American (0.06±0.01 vs 0.07±0.01, for LF vs HF diet, respectively, p=0.40) or in the white women (0.09±0.01 vs 0.10±0.01, for LF vs HF diet, respectively, p=0.09). In most women, insulin sensitivity either remained unchanged or was higher after the HF compared to the LF diet (figure 1A and B). Similarly, metabolic flexibility (ΔNPRQ during the clamp) was not significantly altered by the diet type (table 2).

Using data from all participants, the steady-state insulin levels during the clamp were higher in the African–American vs non-Hispanic white women, after adjustment for the postabsorptive values, after the LF diet (1449.17±40.34 pmol/L vs 1247.48±80.68 pmol/L, p=0.02) or after the HF diet (1490.98±59.59 pmol/L vs 1286.55±103.75 pmol/L, p=0.05). Thus, the calculated insulin clearance was lower in African–American vs white women, after the LF diet (407.55±12.26 mL/m^2^/min vs 489.60±30.19 mL/m^2^/min, p=0.03) or after the HF diet ((397.02±14.27 mL/m^2^/min vs 486.86±34.65 mL/m^2^/min, p=0.04). There were no other significant differences by race, after the LF diet (p range 0.91–1.0) or after the HF diet (p range 0.14–0.9).

Fat oxidation was significantly suppressed by insulin during the euglycemic clamp, for both African–American and white women, after both the LF (p<0.001) and HF (p<0.001) diets (figure 2A), with no significant insulin by race interaction (figure 2B) on either diet (p=0.27 and p=0.28, respectively). ΔNPRQ, that is, metabolic flexibility during the clamp, was not significantly different in African–American vs white women after the LF diet (0.10±0.02 vs 0.12±0.02, respectively, p=0.59) or after the HF diet (0.12±0.02 vs 0.13±0.02, p=0.58).

## Conclusions

Our study did not show a decrease in peripheral insulin sensitivity in response to a short-term (1 week) eucaloric 50% HF diet compared to a 30% LF diet in healthy, overweight, and obese premenopausal African–American and non-Hispanic white women. Metabolic flexibility (ΔNPRQ) was similarly unaffected. The only significant race difference we found was the lower insulin clearance in African–American vs white women, regardless of the diet.

Our results highlight the controversy surrounding the effect of a eucaloric increase in the fat content of a weight-maintaining diet on insulin sensitivity and metabolic flexibility, a precursor of insulin sensitivity. One other study, utilizing FSIVGTT to measure insulin sensitivity in premenopausal obese women showed a deterioration of insulin sensitivity after 3 weeks of a eucaloric HF diet vs a eucaloric LF diet,^13^ whereas other studies, in agreement with our results, have used a euglycemic hyperinsulinemic clamp to assess insulin sensitivity, which is the ‘gold standard’ for this outcome. A eucaloric HF diet consumed over a period of ∼3 weeks did not alter insulin sensitivity in mixed groups of lean men and women,^11^ ^12^ and similar results were demonstrated in lean men after just 6 days,^7^ and in lean and overweight men after 3 weeks,^8^ ^10^ of a eucaloric HF diet. Thus, diet duration does not seem to account for the discrepancy between our results and other work in a similar population.^13^ Hepatic insulin sensitivity remained unchanged in two studies with a similar HF diet as used by us,^7^ ^10^ but was shown to decrease in lean men after 11 days of an 83% HF diet.^9^ FSIVGTT does not differentiate between hepatic and peripheral insulin×sensitivity. Different effects of a HF diet on hepatic vs peripheral insulin sensitivity may to some extent account for the difference in results noted by us.^13^ Other factors playing a role may be the account of the menstrual cycle stage when insulin sensitivity was measured,^13^ ^25^ and the differences in the amounts of saturated fat employed.^13^

We also found that the metabolic flexibility, measured as a suppression of fat oxidation during the hyperinsulinemic (80 mU/m^2^/min) euglycemic clamp (ΔNPRQ),^14^ was not affected by the 1 week of a eucaloric 50% HF diet in our women. The effect of a eucaloric HF diet on ΔNPRQ has been studied in men, yet the results are inconclusive. In lean men, ΔNPRQ was not decreased in response to an HF (75%) diet compared to a similar LF (35%) diet, after 6 days,^7^ or 3 weeks,^10^ but was decreased after 11 days of a HF (83%) diet.^9^ In overweight men, ΔNPRQ decreased after 3 weeks of an HF (55%) diet.^8^ No similar studies are available in women. A certain threshold in the fat/carbohydrate ratio of the diet and the effect on hepatic insulin sensitivity^26^ may modulate the degree of fat oxidation suppression by insulin after a eucaloric HF diet. Hepatic insulin sensitivity and its relationship to metabolic flexibility was not evaluated in our study and needs to be investigated further. Some of the findings in the present study, specifically a lack of differential effect by race, may be due to a lack of power secondary to a small sample size. Furthermore, 1 week of a eucaloric 50% HF diet may have different effects in other populations, with different genetic susceptibility.^27^ ^28^

Finally, we previously reported lower rates of postabsorptive fat oxidation in response to an HF diet and lack of fat oxidation suppression by insulin during a pancreatic clamp in African–American vs white women.^15^ In this study, we observed similar trends for the postabsorptive fat oxidation values, but the higher dose of insulin during the clamp similarly suppressed fat oxidation in the two races, in agreement with a recent report.^29^ We also found lower insulin clearance in the African–American women compared to the white women, in contrast to one,^30^ but in agreement with another study in adult women.^31^ The lower insulin clearance could be contributing to unmeasured postprandial hyperinsulinemia, which may partly explain the numerous reports of lower fat oxidation rates in African–Americans without diabetes compared to other white populations.^21^ ^22^ ^32^ ^33^

In conclusion, peripheral insulin sensitivity was not deleteriously affected by 1 week of a eucaloric HF diet (50% of total Kcal from fat), compared to a LF (30% of total Kcal from fat) diet, in healthy, premenopausal, overweight and obese African–American and non-Hispanic white women. Our findings need to be verified with regard to the effect on hepatic insulin response and more importantly in other susceptible populations.
